# The Effects of *Cosmos caudatus* on Structural Bone Histomorphometry in Ovariectomized Rats

**DOI:** 10.1155/2012/817814

**Published:** 2012-08-08

**Authors:** Norazlina Mohamed, Sharon Gwee Sian Khee, Ahmad Nazrun Shuid, Norliza Muhammad, Farihah Suhaimi, Faizah Othman, Abdul Salam Babji, Ima-Nirwana Soelaiman

**Affiliations:** ^1^Department of Pharmacology, Universiti Kebangsaan Malaysia Medical Center, Jalan Raja Muda Abdul Aziz, 50300 Kuala Lumpur, Malaysia; ^2^Department of Biomedical Sciences, Faculty of Health Sciences, Universiti Kebangsaan Malaysia, Jalan Raja Muda Abdul Aziz, 50300 Kuala Lumpur, Malaysia; ^3^Department of Anatomy, Universiti Kebangsaan Malaysia Medical Center, Jalan Raja Muda Abdul Aziz, 50300 Kuala Lumpur, Malaysia; ^4^School of Chemical Sciences and Food Technology, Faculty of Science and Technology, Universiti Kebangsaan Malaysia, 43600 Bangi, Malaysia

## Abstract

Osteoporosis is considered a serious debilitating disease. *Cosmos caudatus* (*ulam raja*), a plant containing antioxidant compounds and minerals, may be used to treat and prevent osteoporosis. This study determines the effectiveness of *C. caudatus* as bone protective agent in postmenopausal osteoporosis rat model. Thirty-two female rats, aged 3 months old, were divided into 4 groups. Group one was sham operated (sham) while group two was ovariectomized. These two groups were given ionized water by forced feeding. Groups three and four were ovariectomized and given calcium 1% ad libitum and force-fed with *C. caudatus* at the dose of 500 mg/kg, respectively. Treatments were given six days per week for a period of eight weeks. Body weight was monitored every week and structural bone histomorphometry analyses of the femur bones were performed. Ovariectomy decreased trabecular bone volume (BV/TV), decreased trabecular number (Tb.N), and increased trabecular separation (Tb.Sp). Both calcium 1% and 500 mg/kg *C. caudatus* reversed the above structural bone histomorphometric parameters to normal level. *C. caudatus* shows better effect compared to calcium 1% on trabecular number (Tb.N) and trabecular separation (Tb.Sp). Therefore, *Cosmos caudatus* 500 mg/kg has the potential to act as the therapeutic agent to restore bone damage in postmenopausal women.

## 1. Introduction

Estrogen deficiency increases the risk of developing osteoporosis. Estrogen was found to have antioxidant properties [1] and was also shown to increase the expression of glutathione peroxidase in osteoclasts [2], an enzyme which is responsible for the degradation of hydrogen peroxide. Estrogen deficiency will reduce the expression of the enzyme and renders the bone susceptible to hydrogen peroxide attacks.

In osteoporosis, lipid peroxidation is increased due to the reduction in antioxidants [3], and reactive oxygen species are found to play a role in bone metabolism [4]. Free radicals have also been shown to be cytotoxic to osteoblastic cells [5]. Loss of estrogens accelerates the effects of aging on bone by decreasing defence against oxidative stress which leads to bone loss [6].

Since free radicals and lipid peroxidation are involved in bone metabolism and may be the culprit in causing bone loss, substances having antioxidative activities can overcome the detrimental effects. Our previous studies have shown the beneficial effects of palm-oil derived tocotrienols in several experimental osteoporosis, ovariectomized [7], steroid induced [8], and nicotine induced [9]. The effects of palm-oil derived tocotrienols may be attributed to its antioxidative activities.

Thus, in finding alternatives in the treatment of osteoporosis, a local plant, *Cosmos caudatus *or locally known as “ulam raja” (King's salad), is of consideration. Previous study has shown that this plant has antioxidative activities [10]. It contains phenolic compounds that contribute to the color, antioxidant, and anticarcinogenic properties of the plants. For every 100 g of *Cosmos caudatus*, the total phenolic compound is 21.41 mg. It is also found that *Cosmos caudatus *had extremely high antioxidant capacity of about 2,400 mg l ascorbic acid equivalent antioxidant capacity (AEAC) per 100 g of fresh sample [11]. It is also believed that *Cosmos caudatus *promote the formation of healthy bones [12]. Thus, we hypothesized that *Cosmos caudatus *may exert protective effects on bone of ovariectomized rats which is a suitable animal model for studying postmenopausal osteoporosis. In this study, the effects of *Cosmos caudatus *on structural bone histomorphometry were determined.

## 2. Materials and Methods

### 2.1. Animals and Treatment

Thirty-two young adult (3 months) female Wistar rats, weighing 190 g–260 g, were obtained from the Laboratory Animal Resource Unit, Faculty of Medicine, Universiti Kebangsaan Malaysia. Rats were randomly assigned to four groups with eight rats in each group. Group 1 was sham operated (sham) while the second was ovariectomized-control group (OVX). The third and fourth groups were ovariectomized and treated with calcium 1% (Ca) ad libitum and force-fed with 500 mg/kg *C. caudatus* extract (CC), respectively. Treatment was given six days a week for eight weeks and body weight was recorded weekly. The study was approved by the Universiti Kebangsaan Malaysia Animal Ethics Committee with the approval code of PP/FAR/2008/NORAZLINA/12-AUGUST/225-SEPT-2008-AUG-2009.

#### 2.1.1. Diet, *Cosmos caudatus*, and Calcium 1%

All rats received normal rat chow obtained from Gold Coin, Malaysia. The composition of rat chow is shown in Table 1. The aqueous extract of *C. caudatus* with the concentration of 500 g/300 mL was prepared by School of Chemical Sciences & Food Technology, Faculty of Science and Technology, Universiti Kebangsaan Malaysia using water extraction method which was previously described [10]. The 500 mg/kg dose was prepared by mixing *C. caudatus* with deionized water in ratio 3 : 7. Calcium 1% solution was prepared by mixing 1 g of hemicalcium lactic acid (Sigma Chemical CO., USA) with 100 mL deionized water.

### 2.2. Ovariectomy

Before the surgery, the rats were anesthetized with Ketamil and Ilium Xylazil-20 (Troy Laboratories PTY, Australia), given intraperitoneally, at 1 : 1 ratio. A vertical incision was made approximately 15 cm in the abdomen using a sterilized sharp knife. The right and left ovaries were cut and removed. Before the ovaries were cut, the fallopian tubes were tied to prevent bleeding. The muscle layer under the skin was stitched up by sterile and soluble suture (Serafit, Germany). Then, the outer layer of skin was sewn with nonwater soluble suture (Seralon, Serag Wiessner, Germany). The procedure for sham operated rats was just the same with ovariectomized rats, but both of the ovaries were not removed. Rats were left recuperating for 1 week before commencing the treatment.

### 2.3. Bone Histomorphometry

Upon sacrifice, the distal part of the femur was fixed with 70% ethanol and undergoes undecalcified bone preparations. The bone samples were embedded in polymer methyl methacrylate according to Difford [13], sectioned at 9 *μ*m thickness using a microtome, stained using von Kossa method [14], and analyzed using an image analyzer with the Video Test-Master software. The parameters measured were trabecular bone volume (BV/TV), trabecular thickness (Tb.Th), trabecular number (Tb.N), and trabecular separation (Tb.Sp). All measurements were performed randomly at the metaphyseal region, which was located 3–7 mm from the lowest point of the growth plate and 1 mm from the lateral cortex [15]. The selected area is the secondary spongiosa area, which is rich in trabecular bone. All parameters were measured according to the American Society of Bone Mineral Research Histomorphometry Nomenclature Committee [16].

### 2.4. Statistical Analysis

Results were presented as mean ± standard error of the mean (SEM). All data were analysed using the Statistical Package for Social Sciences software. The Kolmogorov-Smirnov test was used for normality. ANOVA followed by Tukey's HSD tests were used for normally distributed data while Kruskal-Wallis and Mann-Whitney tests were used for not normally distributed data.

## 3. Results

**Figure 1:**
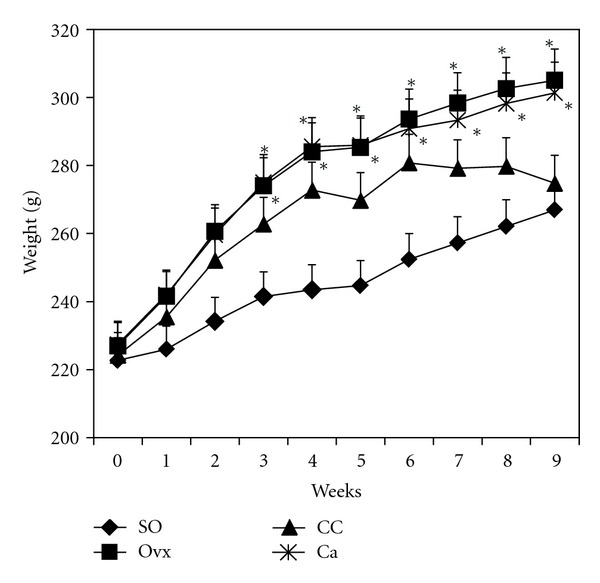
Body weight of rats for all treatment groups. *Indicates significant difference (*P* < 0.05) compared to SO group. SO, sham operated; Ovx, ovariectomized; CC, ovariectomized and supplemented with 500 mg/kg *Cosmos caudatus*; Ca, ovariectomized and supplemented with 1% calcium.

Figure 1 shows the body weight of all groups throughout the study period. Ovariectomized group gained weight and was significantly different compared to sham-operated group beginning from week 3. Similar findings were observed in the calcium supplemented group. Group supplemented with *C. caudatus *also showed weight gain but did not differ compared to the sham-operated group.

**Figure 2:**
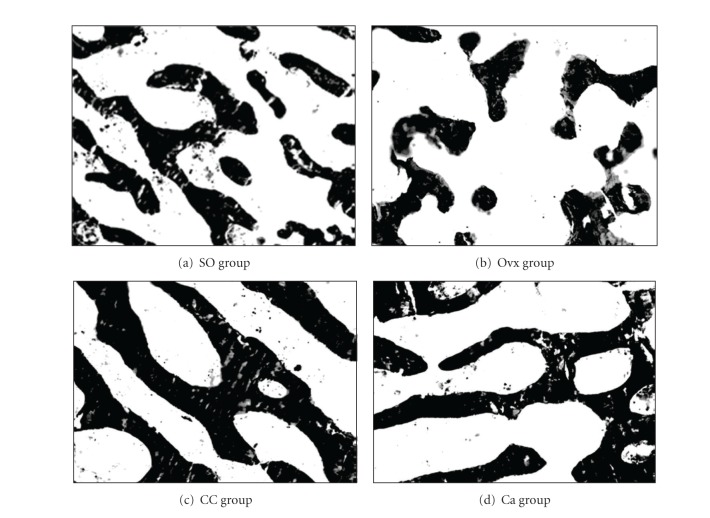
Photomicrographs of trabecular bone. Undecalcified section (100 x magnification) shows trabecular bone (stained black) using von Kossa method. SO, sham operated; Ovx, ovariectomized; CC, ovariectomized and supplemented with 500 mg/kg *Cosmos caudatus*; Ca, ovariectomized and supplemented with 1% calcium.

**Figure 3:**
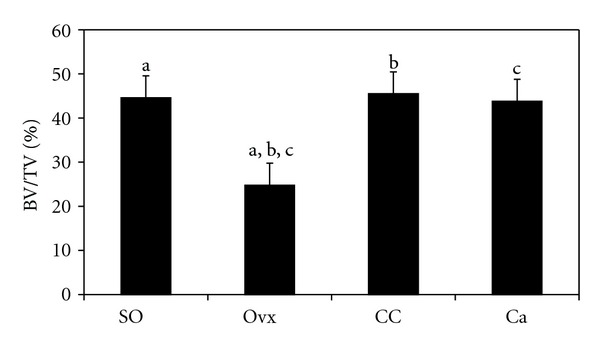
Effects of *Cosmos caudatus* supplementation on bone volume in ovariectomized rats. Groups which share the same alphabet indicate significant difference (*P* < 0.05). SO, sham operated; Ovx, ovariectomized; CC, ovariectomized and supplemented with 500 mg/kg *Cosmos caudatus*; Ca, ovariectomized and supplemented with 1% calcium.

**Figure 4:**
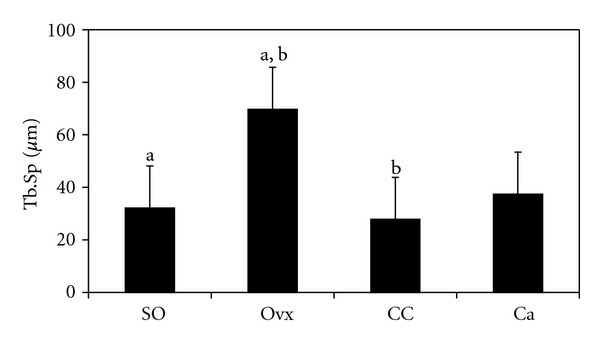
Effects of *Cosmos caudatus *supplementation on trabecular separation in ovariectomized rats. Groups which share the same alphabet indicate significant difference (*P* < 0.05). SO, sham operated; Ovx, ovariectomized; CC, ovariectomized and supplemented with 500 mg/kg *Cosmos caudatus*; Ca, ovariectomized and supplemented with 1% calcium.

**Figure 5:**
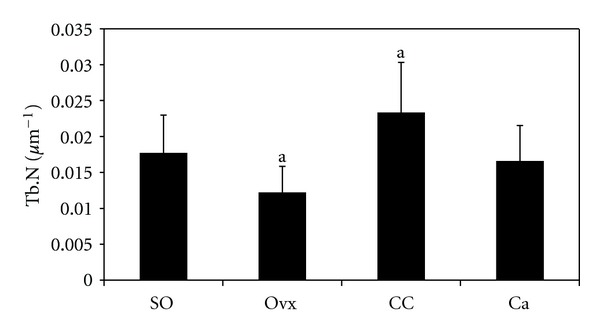
Effects of *Cosmos caudatus *supplementation on trabecular number in ovariectomized rats. Groups which share the same alphabet indicate significant difference (*P* < 0.05). SO, sham operated; Ovx, ovariectomized; CC, ovariectomized and supplemented with 500 mg/kg *Cosmos caudatus*; Ca, ovariectomized and supplemented with 1% calcium.

**Figure 6:**
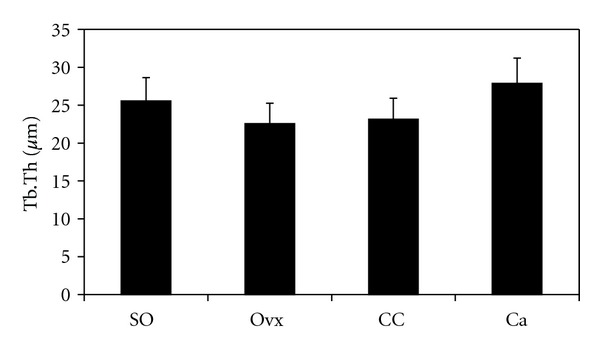
Effects of *Cosmos caudatus *supplementation on trabecular thickness in ovariectomized rats. SO, sham operated; Ovx, ovariectomized; CC, ovariectomized and supplemented with 500 mg/kg *Cosmos caudatus*; Ca, ovariectomized and supplemented with 1% calcium.

Figure 2 shows the photomicrographs of trabecular bone with von Kossa staining for the different treatment groups. Ovariectomy caused a reduction in bone volume (BV/TV) and an increase in trabecular separation (Tb.Sp) compared to the control group (Figures 3 and 4, resp.). Administration of *C. caudatus* was able to improve bone volume and trabecular separation in ovariectomized rats (Figures 3 and 4, resp.) while calcium administration was only able to improve bone volume (Figure 3). In addition, the group supplemented with *C. caudatus* showed a higher trabecular number compared to ovariectomized group (Figure 5). No significant differences were seen in trabecular thickness (Figure 6).

## 4. Discussion

Ovariectomized rats have been used by researchers as the model for postmenopausal osteoporosis. Even though limitations exist, certain characteristics in the rat model mimic the bone changes in postmenopausal women and made the study of the human disease possible [17, 18]. Reduction in bone mineral density occurs two months after ovariectomy with greater loss seen in regions rich in trabecular bone [19].

Ovariectomized group showed increase in body weight which was statistically different compared to sham-operated group beginning from week 3 (Figure 1). This observation is common in estrogen-deficient animals since the deficient state induces hyperphagia in rats [20]. Group supplemented with calcium showed the same findings. However, rats given *C. caudatus* did not show a significant increase in body weight. *Cosmos caudatus *seemed able to prevent weight gain induced by ovariectomy. However, the exact mechanism of this occurrence is unknown.

In this study, ovariectomy caused loss of bone volume, increase in trabecular separation, and decrease in trabecular number (Figures 3, 4, and 5, resp.). The bone loss is reflected in the photomicrograph of the trabecular bone in which the ovariectomized group showed perforated and discontinued trabecular bone compared to the sham-operated group (Figures 2(a) and 2(b)). Similar findings were observed in other studies [21, 22]. However, trabecular thickness did not show any significant differences between the different groups. This is in contrast with other studies [23, 24] which used micro-CT in their studies as opposed to conventional histomorphometry in our study. In another study which used older rats, ovariectomy was also shown to cause a decrease in trabecular thickness [25]. Different method and different age range of the animals may contribute to the discrepancy seen in this study.

Estrogen deficiency is the major factor which affects bone in ovariectomized rats [26]. The deficiency state induces osteocytes apoptosis which further leads to increase in osteoclastic resorption [27]. Ovariectomy has been associated with increase in oxidative stress as evident by high malondialdehyde levels [28]. The condition of oxidative stress would eventually lead to bone loss [29].

Ovariectomized rats supplemented with *C. caudatus* showed improvements in bone volume, trabecular separation and trabecular number (Figures 3, 4, and 5, resp.). Photomicrograph of the trabecular bone of rats given *C. caudatus* appears similar to the sham-operated rats (Figures 2(a) and 2(c)). *Cosmos caudatus *is shown to contain flavonoids [30] and ascorbic acid [31]. It has also been shown to exert antioxidant activity [32]. This may contribute to the bone protective effects of *C. caudatus* observed in the present study.

The dose of *C. caudatus *in this study was chosen based on previous study which used *C. caudatus* at the dose of 100, 200, and 300 mg/kg. In the previous study, the effects of *C. caudatus *on bone biochemical markers and bone histomorphometry in ovariectomized rats were determined. It was found that *C. caudatus *at all doses was able to prevent the increase in interleukin-1 and pyridinoline as seen in the ovariectomized group. However, no significant changes were seen in the bone histomorphometry parameters (unpublished data). Thus, in the present study, a higher dose, 500 mg/kg, was used.

In an acute toxicity study done previously, a single dose of *C. caudatus* was given to male rats at the dose of 50, 500, and 2000 mg/kg. The rats were kept for 7 days before sacrifice. The higher dose of *C. caudatus*, that is, 2000 mg/kg, was found to increase liver enzymes but did not cause any changes on the haematological parameters such as clotting time, bleeding time, platelet levels, and white cell count (unpublished data). The observations above imply that *C. caudatus *at the dose of 500 mg/kg is not associated with side effects.

Calcium supplement in combination with vitamin D is recommended in the treatment regime for patients with osteoporosis [33] to reduce risk of nonvertebral fractures [34]. This combination therapy is considered essential but not sufficient for the treatment of osteoporosis and additional benefit may be obtained with the addition of antiresorptive or anabolic agent [35]. In the present study, calcium supplementation also reversed the effects of ovariectomy on bone volume (Figure 2) but failed to show significant changes in the other parameters. Similar findings were observed in previous study in which ovariectomized rats given calcium supplementation still had lower bone volume and trabecular number as compared to sham-operated rats [36]. In another study using ovariectomized rats, calcium supplementation is shown to improve bone fracture healing but failed to improve strength [37]. Another study showed that calcium supplementation suppresses bone formation in magnesium-deficient rats [38]. The findings above suggested that calcium may need to be present with other elements to exert beneficial effects on bone.

All rats received rat chow which contained 0.8–1.2% calcium. The Ca group received additional 1% calcium in drinking water. However, since the calcium was administered via drinking water, the amount of calcium ingested by the rats may vary between individual rats thus causing inconsistent results in the histomorphometry parameters mentioned above.

In the present study, we observed that *C. caudatus* improved bone structural histomorphometric parameters. In fact, *C. caudatus* seemed to yield better effects on bone in terms of trabecular separation and trabecular number (Figures 4 and 5). According to Nutriweb Malaysia [39], *C. caudatus* contains 270 mg calcium per 100 g of the plant. This composition, in addition to its antioxidative property, enables *C. caudatus* to have a greater effect in reversing bone changes induced by ovariectomy as opposed to calcium supplementation.

Further studies are required to establish the effects of *Cosmos caudatus *on bone such as the effects on dynamic histomorphometry, bone biomarkers, bone density, and bone calcium content. In addition, further studies are also required to ascertain the active compound and calcium content of the plant as well as its exact mechanism on bone.

## 5. Conclusion

*Cosmos caudatus *at the dose of 500 mg/kg reversed bone changes induced by ovariectomy. Thus, *C. caudatus *has the potential to be used as an alternative for the treatment of postmenopausal osteoporosis.

## Figures and Tables

**Table 1:** Composition of rat chow (Golden Coin, Malaysia).

Composition	Amount/percentage
Crude protein	21–23%
Crude fibre (max)	5.0%
Crude fat (min)	3.0%
Moisture (max)	3.0%
Calcium	0.8–1.2%
Phosphorus	0.6–1.0%
Nitrogen free extract	49.0%
Vitamin A	10 M.I.U.
Vitamin D_3_	2.5 M.I.U
Vitamin E	15 g
Vitamin K	trace
Vitamin B_12_	trace
Thiamine	trace
Riboflavin	trace
Pantothenic acid	trace
Niacin	trace
Pyridoxine	trace
Choline	trace
Santoquin	trace
Microminerals	trace
